# Are Raw *Brassica* Vegetables Healthier Than Cooked Ones? A Randomized, Controlled Crossover Intervention Trial on the Health-Promoting Potential of Ethiopian Kale

**DOI:** 10.3390/nu10111622

**Published:** 2018-11-02

**Authors:** Nina Schlotz, Grace A. Odongo, Corinna Herz, Hanna Waßmer, Carla Kühn, Franziska S. Hanschen, Susanne Neugart, Nadine Binder, Benard Ngwene, Monika Schreiner, Sascha Rohn, Evelyn Lamy

**Affiliations:** 1 Molecular Preventive Medicine, Institute for Infection Prevention and Epidemiology, Faculty of Medicine and Medical Center, University of Freiburg, 79106 Freiburg, Germany; nina.schlotz@uni-konstanz.de (N.S.); grace.odongo@uniklinik-freiburg.de (G.A.O.); corinna.herz@uniklinik-freiburg.de (C.H.); hanna.wassmer@uiklinik-freiburg.de (H.W.); 2 Institute of Food Chemistry, Hamburg School of Food Science, University of Hamburg, 20146 Hamburg, Germany; carla.kuehn@chemie.uni-hamburg.de (C.K.); rohn@chemie.uni-hamburg.de (S.R.); 3 Leibniz Institute of Vegetable and Ornamental Crops, 14979 Großbeeren, Germany; hanschen@igzev.de (F.S.H.); neugart@igzev.de (S.N.); Benard.Ngwene@agcocorp.com (B.N.); schreiner@igzev.de (M.S.); 4 Institute for Prevention and Cancer Epidemiology, Faculty of Medicine and Medical Center, University of Freiburg, 79106 Freiburg, Germany; nadine.binder@uniklinik-freiburg.de

**Keywords:** aflatoxin B1, *Brassica* vegetables, cancer chemoprevention, anti-genotoxicity, comet assay

## Abstract

The present human intervention trial investigated the health-promoting potential of *B. carinata,* with a focus on effects of thermal processing on bioactivity. Twenty-two healthy subjects consumed a *B. carinata* preparation from raw (allyl isothiocyanate-containing) or cooked (no allyl isothiocyanate) leaves for five days in a randomized crossover design. Peripheral blood mononuclear cells were exposed to aflatoxin B1 (AFB1), with or without metabolic activation using human S9 mix, and subsequently analyzed for DNA damage using the comet assay. Plasma was analyzed for total antioxidant capacity and prostaglandin E_2_ (PGE_2_) levels. Cooked *B. carinata* significantly reduced DNA damage induced by AFB1 as compared to baseline levels (+S9 mix: 35%, −S9 mix: 33%, *p* ≤ 0.01, respectively). Raw *B. carinata* only reduced DNA damage by S9-activated AFB1 by 21% (*p* = 0.08). PGE_2_ plasma levels were significantly reduced in subjects after consuming raw *B. carinata*. No changes in plasma antioxidant capacity were detectable. A balanced diet, including raw and cooked *Brassica* vegetables, might be suited to fully exploit the health-promoting potential. These results also advocate the promotion of *B. carinata* cultivation in Eastern Africa as a measure to combat effects of unavoidable aflatoxin exposure.

## 1. Introduction

Certain fruits and vegetables used for human nutrition are especially beneficial for human health due to their comparatively high amounts of bioactive compounds. Among those, the *Brassicaceae* family comprises some of the best studied and most widely consumed foods with potential health-promoting properties. Prominent examples are vegetables of the genus *Brassica*, such as broccoli or kale. Epidemiological studies revealed a negative correlation between the frequency of consumption of *Brassica* vegetables and the incidence of various cancers [1,2]. The cancer preventive effects are attributed to certain bioactive compounds in the plants, mainly to glucosinolates (GLS) and some of their breakdown products, the isothiocyanates (ITC) [3]. *Both* in vitro and in vivo studies showed that *Brassicaceae* species can improve detoxification of carcinogens and alleviate oxidative stress or inflammation. Studies with isolated or synthesized ITC further support hypotheses that these reactive small molecules account for the anti-genotoxic, antioxidant, or anti-inflammatory activity observed [4]. Moreover, *Brassicaceae* are rich in polyphenols, which are also well known for their cancer preventive potential [5].

*Brassica carinata*, or Ethiopian kale, originates from Ethiopia where it is cultivated as an oil seed and leafy vegetable plant. A rising trend in the use and popularity of *B. carinata* can be observed over the last years. Leaves and seeds are highly rich in nutrients and are reported to contain high concentrations of the GLS sinigrin as well as polyphenols [6,7]. The ITC formed from sinigrin—allyl ITC (AITC)—exhibits many desirable attributes of a chemopreventive agent [8], and recent in vitro results revealed protective effects of *B. carinata* extracts against the mycotoxin aflatoxin B1 (AFB1) in liver cancer cells [9].

AFB1 is the most potent naturally occurring chemical liver carcinogen [10,11], potentially causing both acute toxicity (aflatoxicosis) and chronic diseases [12]. Chronic dietary exposure to AFB1 is a significant risk for the formation of hepatocellular carcinoma (HCC) [13]. Populations around the world are frequently exposed to largely uncontrolled amounts of AFB1, and exposure via contaminated food is quite common and difficult to prevent [14]. Finding effective prevention against AFB1-induced cancer remains a challenging task of high importance [15]. The possibility to use vegetables, like *B. carinata*, for health-promoting purposes is intriguing because they have adapted to adverse conditions, are pest-resistant, and easy to cultivate, highlighting their potential for agricultural and horticultural use worldwide.

Leaves and tender stems of *B. carinata* can be eaten raw, boiled, or pickled. Many plant foods from the *Brassicaceae* as well as other African leafy vegetables (ALVs) are characterized by bitter taste when eaten raw [16,17]. For this reason, they are often preferred cooked [18]. This thermal processing reduces bitterness. However, certain cooking practices, such as extensive cooking periods, might affect the phytochemical constituents, their bioactivity, and, in consequence, the health benefits of consumption. Acknowledging ITC as the plant bioactive predominantly responsible for the chemopreventive effects described in previous studies, one would have to assume that cooking might substantially reduce health benefits because the heat-induced myrosinase inactivation reduces ITC formation [3]. The extent of ITC loss, however, might strongly depend on the duration and processing method [19].

The present intervention trial was conducted (I) to assess the health promoting potential of raw versus cooked *B. carinata* leaves in humans, (II) to determine whether AITC is involved in the observed effects, and, consequently, (III) make conclusions about the commonly assumed adverse effects of cooking for health benefits of *Brassica* vegetable consumption.

## 2. Materials and Methods

### 2.1. Chemicals

Ex vivo and in vitro experiments: Roswell Park Memorial Institute (RPMI) 1640 medium, fetal calf serum (FCS), phosphate buffered saline (PBS, without Ca and Mg), _L_-glutamine, and penicillin/streptomycin (P/S) solution were purchased from Gibco™, Life Technologies GmbH (Darmstadt, Germany). Dimethyl sulfoxide (DMSO; purity > 99%) was purchased from Applichem GmbH (Darmstadt, Germany). Aflatoxin B1 (AFB1, isolated from *Aspergillus flavus*; purity ≥ 98%), arylsulfatase (isolated from *Helix pomatia*), β-nicotinamide adenine dinucleotide phosphate hydrate (NADP), glucose-6-phophate dehydrogenase (G6PDH), absolute ethanol (EtOH), hydrochloric acid (37%), trypan blue, ethidium bromide, N-acetyl–_L_-cysteine (NAC), DEAE-Sephadex A-25, magnesium chloride (MgCl_2_), glucose-6-phosphate (G6P), and hydrogen peroxide (H_2_O_2_) 30% (*w*/*w*) solution in water were purchased from Sigma-Aldrich Chemie GmbH (Taufkirchen, Germany). Pooled human liver S9 fractions were purchased from ThermoFisher Scientific (Darmstadt, Germany). Low melting point agarose (LMPA) and normal melting point agarose (NMPA) were purchased from Serva GmbH (Heidelberg, Germany).

Metabolite analyses in plasma and urine: Trifluoracetic acid (TFA, 99.5%) was obtained from AppliChem GmbH (Darmstadt, Germany), formic acid (FA, 98%) was obtained from Carl Roth GmbH & Co. KG (Karlsruhe, Germany), and potassium chloride (KCl) and methanol (MeOH) of ultra LC-MS grade was purchased from Merck KG (Darmstadt, Germany). All aqueous solutions were prepared with ultrapure deionized water. C18ec SPE cartridges (3 mL, 200 mg) were purchased from Macherey-Nagel (Düren, Germany). Aphidicolin was purchased from Cayman Chemical (Ann Arbor, MI, USA).

Chemical analyses of the plant material: allyl isothiocyanate (AITC; ≥99%), and benzonitrile (≥99.9%) were purchased from Sigma-Aldrich Chemie GmbH (Taufkirchen, Germany). Methylene chloride (GC Ultra Grade), chlorogenic acid, quercetin 3-*O*-glucoside, kaempferol 3-*O*-glucoside, and isorhamnetin-3-*O*-glucoside were from Carl Roth GmbH and Co. KG (Karlsruhe, Germany).

### 2.2. Participants

**Figure 1:**
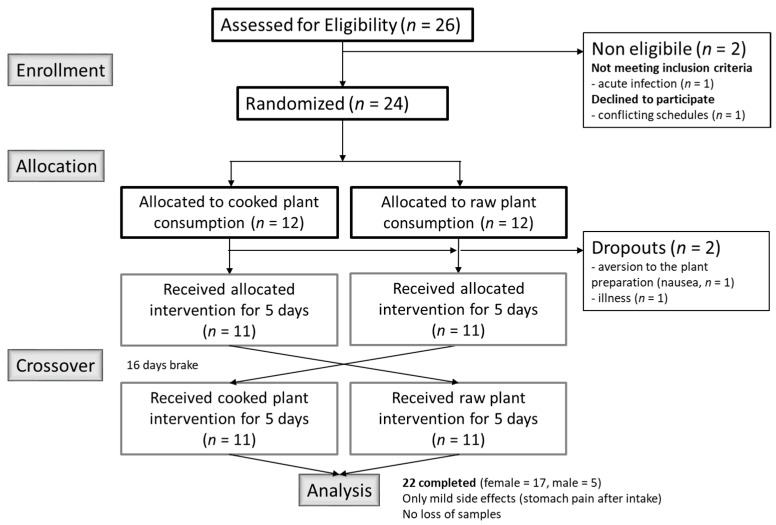
Flow diagram of the randomized controlled cross-over intervention trial with *B. carinata*.

A total of 26 healthy subjects (5 males, 21 females) were recruited via posted flyers, social media, and personal communication in Freiburg im Breisgau, Germany. After providing written informed consent, subjects were screened for suitability using a health questionnaire. The criteria for qualifying for the study included age between 18 to 40 years, good health status, and non-smoking. Exclusion criteria included: Acute and chronic diseases, severe allergies, pregnancy, and strong over- or under-weight (Body Mass Index (BMI) >26 or <18). Two female subjects dropped out before randomization: One for health reasons (developed fever before randomization), and one had conflicting schedules with planned study. During allocation, two more female participants dropped out: One due to acute illness and one due to a strong aversion regarding the *B. carinata* preparation (nausea after intake). Twenty-two participants (5 males, 17 females) completed the study. Only mild side effects (stomach pain after intake) were reported during the study. The study was conducted according to the Declaration of Helsinki and all procedures involving human subjects were approved by the Ethics Committee of the University of Freiburg (ethical vote number 277/16). This trial was registered with the German Clinical Trials Register (DRKS; ID: DRKS00010836). The study complies with the CONSORT Statement for randomized trials (Figure 1).

### 2.3. Brassica carinata Leaf Preparation

The *B. carinata* leaf preparation was prepared freshly every day prior to intake by the subjects. 15 g of freeze-dried, ground *B. carinata* leaves were thoroughly suspended in 200 mL drinking water in closed glass bottles and incubated for 1 h at room temperature. Bottles were shaken occasionally. Immediately before consumption, a small amount of refined sugar was added and dissolved. Two different preparations were used for the trial: (1) A preparation from raw, unprocessed leaves, freeze-dried directly after harvesting; the enzyme, myrosinase, was intact and ITC were produced during incubation of the preparation (=active preparation). (2) A preparation from the freeze-dried leaves, which had been cooked for 10 min (200 g ad 4 L of boiling water), cooled immediately, and freeze dried again. With this procedure, the myrosinase was inactivated and no ITC were formed during incubation of the preparation (=inactivated preparation). Consequently, the preparation from raw *B. carinata* leaves contained 177 µmoL AITC per serving and 6 µmoL residual sinigrin, whereas the preparation from cooked *B. carinata* leaves contained 269 µmoL sinigrin per serving and did not contain AITC, as analyzed using the method of Hanschen et al. [20]. AITC was quantified relative to the internal standard benzonitrile and the response factor of AITC relative to benzonitrile was determined using standard curves of authentic standards [20]. The cooking procedure reduced the concentration of acylated quercetin and kaempferol tri and tetraglycosides while non-acylated kampherol-3-sophoroside-7-glucoside was increased as described earlier [7]. The cooking procedure did not significantly change the total polyphenol content (Supplementary Table S1). Polyphenols were analyzed using the method of Odongo et al. [21].

### 2.4. Study Design and Protocol

**Figure 2:**
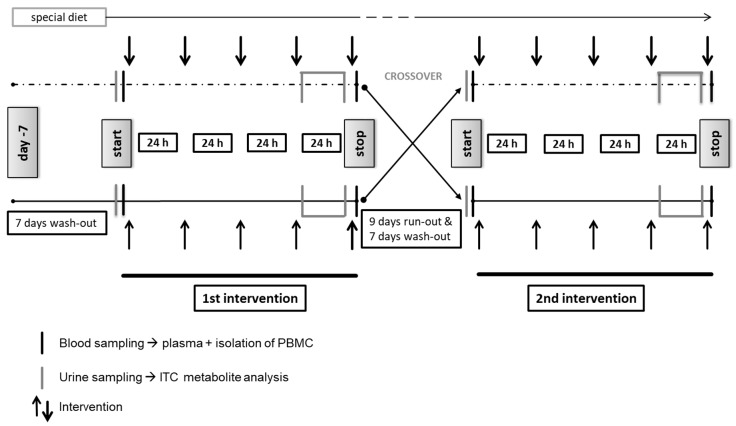
Study design of the randomized controlled cross-over intervention trial with *B. carinata*.

A randomized, single blind (only participants were blinded), controlled crossover trial was conducted (for study design see Figure 2) at the Medical Center of the University of Freiburg, Germany. Recruitment started on 5 September 2016; the last intervention phase ended on 18 November 2016. Randomization was done using Microsoft^®^ Excel. Each subject attended two 5-day intervention phases separated by a 9-day run-out phase. Both intervention phases were preceded by a 7-day wash-out period and accompanied by a special GLS/ITC-free and polyphenol-reduced diet (Supplementary Table S2). During each of the 5-day interventions, the subjects consumed one serving of the respective *B. carinata* preparation every morning, either the active or the inactivated preparation. The sequence of consumption (start with active or inactivated preparation) was assigned randomly by one of the investigators.

The full trial protocol can be accessed at the German Clinical Trials Register (DRKS; ID: DRKS00010836).

### 2.5. Collection and Preparation of Blood and Urine Samples

On day one, subjects arrived fasting at the study center. They were asked to provide a spontaneous urine sample, which was immediately placed on ice. Then, the initial blood sample (before intake of the *B. carinata* preparation) was drawn into Li-Heparin and ethylenediaminetetraacetic acid (EDTA) vacutainers by venipuncture. EDTA vacutainers for plasma generation were placed on ice; Li-Heparin vacutainers for PBMC isolation were kept at room temperature. All samples were processed within 2 h. Subjects collected 24-h urine from day 4 to 5 in 2L urine collection bottles (Sarstedt, AG, Nümbrecht, Germany). On day five, the last *B. carinata* preparation was consumed and the final blood sample was drawn 2 h afterwards (same procedure as for day one, subjects again arrived fasting). This time point was chosen because concentrations of ITC metabolites in human plasma reach a maximum between 2 and 4 h after consumption [22,23]. The subjects were not allowed to eat during the 2 h period.

Urine samples were stored in urine vacutainers at −80°C until chemical analysis. EDTA blood was centrifuged at 4 °C and 2000× *g* for 10 min. The upper plasma layer was separated carefully and aliquots were stored at −80 °C until further analysis.

### 2.6. Isolation of Peripheral Blood Mononuclear Cells (PBMC)

PBMC were isolated from Li-heparinized blood by centrifugation on a LymphoPrep^™^ gradient using SepMate^™^ centrifugation tubes (Stemcell Technologies, Cologne, Germany) and washed twice with PBS. Cell viability and cell concentration were determined using the trypan blue exclusion test.

### 2.7. Comet Assay

The alkaline comet assay for detecting changes of susceptibility of PBMC to AFB1-induced DNA damage was performed according to Lamy et al. [24], with few modifications. After blood sampling on day 1 and day 5 of the intervention, isolated PBMC were exposed to 10 µM AFB1 or 0.1% DMSO in the absence or presence of a metabolic activation system using human S9 microsomes for 4 h as described by van Leeuwen et al. [25]. Then, cells were harvested and prepared for the comet assay analysis. Analysis was done with a Leica fluorescence microscope (Leica DMLS; excitation filter; BP 546/10 nm; barrier filter: 590 nm) connected to an image analysis system (Comet 5.5, Optilas GmbH, München, Germany), with 100 cells per slide being systematically scored. The indicator of DNA damage was % tail DNA (fold to DMSO-treated cells, new baseline for each ex vivo experiment).

### 2.8. Effect of N-acetyl-l-cysteine (NAC) In Vitro

To investigate the effect of the free radical scavenger, NAC, on AFB1-induced DNA damage in PBMC, PBMC were isolated from blood samples of three volunteers. PBMC were pretreated with 5 mM NAC for 1 h and consecutively exposed to 10 µM AFB1 with or without metabolic activation by human S9 microsomes. Following this, the comet assay was performed as described above.

### 2.9. Plasma Total Antioxidant Capacity

The total antioxidant capacity (TAC) was measured in EDTA-plasma using the ImAnOx^®^ (TAS/TAC) Kit (Immundiagnostik AG, Bensheim, Germany) according to the manufacturer’s protocol.

### 2.10. Measurement of AITC Metabolites in Plasma and Urine

AITC metabolites (AITC-glutathione, AITC-GSH; AITC-cysteinylglycine, AITC-CysGly; AITC-cysteine, AITC-Cys; AITC-*N*-acetyl-l-cysteine, AITC-NAC) in plasma and urine were analyzed using LC-ESI-MS/MS analysis. Sample preparation was performed following the procedure described by Platz et al. [22]. LC-ESI-MS/MS analysis was carried out on a 4000 QTrap (AB Sciex Germany GmbH, Darmstadt, Germany) equipped with an Agilent 1200 series HPLC system (Agilent Technologies Deutschland, GmbH & Co. KG, Waldbronn, Germany). The data were acquired and processed by software Application Analyst 1.6.1 (AB SCIEX, Concord, ON, Canada). The analysis was performed on a Phenomenex Kinetex C18 column (5 µm, 100 Å, 150 × 2.1 mm) equipped with a guard column (C18, 2.1 × 4.6 mm). The column was equilibrated in a column oven to 20 °C and the autosampler was maintained at 4 °C. The mobile phase consisted of 0.1% formic acid in water (A) and 0.1% formic acid in methanol (B). The flow rate was set to 250 µL/min and the injection volume was 4 µL. The gradient started with 90% of A and held for 3 min. After that, B was raised from 10% to 90% within 8 min and held at 90% B for 6 min. After that, the column was equilibrated at 10% B for 8 min. The quantitation of the metabolites was done by an external calibration curve in a concentration range between 0.01 µM and 50 µM.

### 2.11. Measurement of Prostaglandin E_2_ (PGE_2_) in Plasma

The PGE_2_ concentration was measured in EDTA-plasma using the Prostaglandin E_2_ ELISA Kit- Monoclonal (Cayman Chemicals, Hamburg, Germany) according to the manufacturer’s protocol. Plasma was diluted 1:2 in ELISA buffer and assayed in duplicate.

### 2.12. Power Calculation and Statistical Analysis

The number of subjects was determined by sample size calculation indicating that 20 subjects would be required for detecting an effect of 25% difference in % tail DNA (pre vs. post intervention) in the response to matched pairs in the crossover study with 20% standard deviation at 90% power and α = 0.01 significance. Assuming a drop-out-rate of 20%, 24 subjects were recruited for the trial. The number of subjects is consistent with other studies determining DNA damage frequency by single cell gel electrophoresis [26,27]. For assessing possible differences in % tail DNA in the active or control intervention group as well as between post and pre values of the active and control group, a non-parametric Wilcoxon test was used after graphically determining variance heterogeneity in the groups. Significant intra- and intergroup changes were indicated by *p* ≤ 0.05. Data derived from the bioassays were analyzed using the *R* statistical environment ([28], version 3.1.2). Data derived from chemical analyses were analyzed using ANOVA with post-hoc Tukey Test.

## 3. Results

### 3.1. General Information

Participants (*n* = 22) were aged 22.7 ± 2.4 years, with a body weight of 70 ± 10.6 kg, height of 1.7 ± 0.1 m, and with a BMI of 23 ± 1.9 kg/m^2^.

### 3.2. Traceability of AITC Metabolites in Plasma and Urine

No AITC-metabolites were detected in plasma or urine samples before intervention start in all subjects, which confirmed the efficacy of the wash-out periods and volunteer compliance to the dietary plan. AITC metabolites were not detected in plasma and urine samples of any subjects that consumed the preparation from cooked (‘inactivated’) *B. carinata* leaves, except for the presence of AITC-NAC, which was detected in urine samples in very low amounts (Table 1). In contrast, different AITC metabolites from the mercapturic acid pathway were detected in the plasma of subjects that consumed the preparation from raw *B. carinata* leaves, but only AITC-NAC was detected in the urine sampled 2 h after the last consumption. Here, AITC-NAC levels were approximately four-fold higher compared to levels observed in subjects that consumed the preparation from cooked *B. carinata* material (Table 1).

### 3.3. Protection Against DNA Damage by B. carinata Intervention

**Figure 3:**
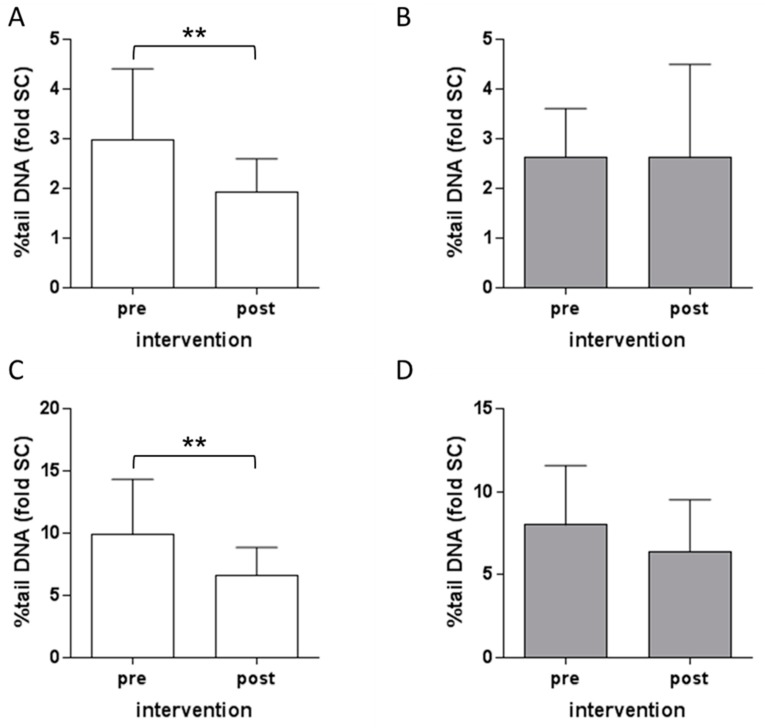
Effect of intervention with *B. carinata* leaf preparation on DNA damage induction by aflatoxin B1 (AFB1). PBMC were exposed to 10 µM AFB1 for 4 h without (**A**,**B**) or in the presence of a metabolic activation system (**C**,**D**) before (pre) and after (post) the intervention with *B. carinata*. White bars show effects of the intervention with preparations from cooked *B. carinata* leaves (−ITC), grey bars show effects of the intervention with preparations from raw *B. carinata* leaves (+ITC) as fold of the solvent control (SC = 0.1% DMSO). Data are means ± SD (*n* = 22). Asterisks indicate statistically significant differences (** *p* ≤ 0.01).

The DNA damage in PBMC induced by AFB1 ex vivo was analyzed using the comet assay. Intervention with the preparation from cooked *B. carinata* leaves resulted in a significant reduction in DNA damage both in the presence and absence of metabolic activation by human S9 mix (Figure 3A,C). Intervention with the preparation from raw *B. carinata* leaves did not show a significant reduction in DNA damage (Figure 3B,D).

### 3.4. Impact of NAC Pretreatment on AFB1-Induced DNA Damage In Vitro

**Figure 4:**
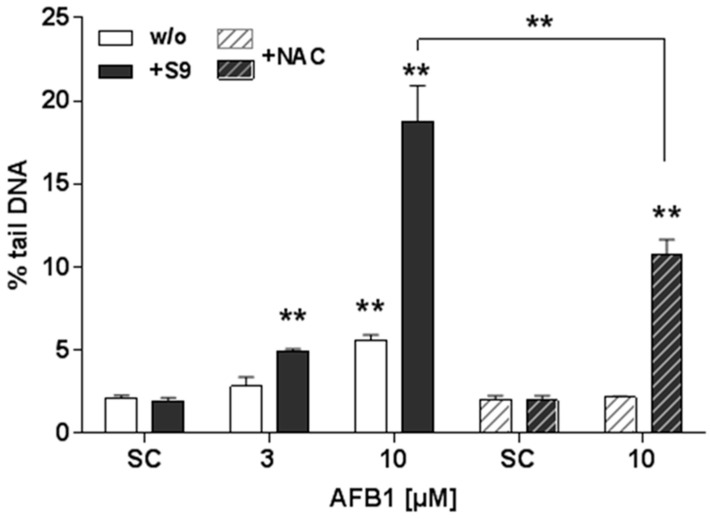
Effects of N-acetylcysteine (NAC) pretreatment on aflatoxin B1 (AFB1)-induced DNA damage. Peripheral blood mononuclear cells (PBMC) were exposed to AFB1 for 4 h without (white bars) or in the presence of S9 mix (black bars) without or with (hashed bars) NAC pre-treatment for 1 h. (SC: Solvent control = 0.1% DMSO). Data are means ± SD (*n* = 3). Asterisks indicate statistically significant difference from the respective SC or between treatments indicated (** *p* ≤ 0.01).

To gain insight into the relevance of oxidative stress for DNA damage induction by AFB1, pre-treatment of isolated PBMC with the antioxidant NAC was used in an accompanying in vitro experiment. Exposure of PBMC to AFB1 concentration-dependently induced DNA damage, in the absence (white bars) and presence (black bars) of human S9 mix (Figure 4). A four-fold increase in DNA damage by 10 µM AFB1 was observed after S9 mix activation as compared to that in cells treated with AFB1 without metabolic activation. When cells were pre-treated with 1 mM NAC, the DNA damage induced by AFB1 was completely inhibited, and the damage induced by AFB1 + S9 mix was significantly reduced by 43 % (Figure 4).

### 3.5. Influence of B. carinata Intervention on Plasma Total Antioxidant Capacity

Intervention with *B. carinata* did not impact the total antioxidant capacity of the subjects’ plasma irrespective of the type of preparation consumed. Mean TAC values for the cooked preparation were 286 ± 37 µmol/L (pre intervention) and 286 ± 38 µmol/L (post intervention). For the raw preparation, mean values were 280 ± 35 µmol/L (pre intervention) and 284 ± 36 µmol/L (post intervention). The values were in the range of a medium antioxidant capacity as stated by the assay kit manufacturer.

### 3.6. Influence of B. carinata Intervention on Prostaglandin E_2_ (PGE_2_) Plasma Levels

**Figure 5:**
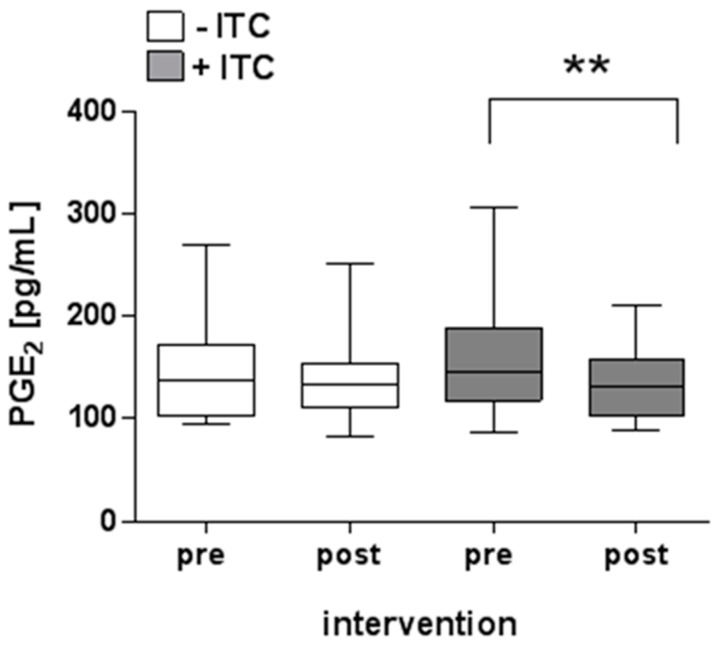
Effects of *B. carinata* intervention on prostaglandin E_2_ (PGE_2_) plasma levels. White boxes show the PGE_2_ plasma concentration before (pre) and after (post) intervention with the preparation from cooked *B. carinata* leaves (−ITC), and grey boxes show the PGE_2_ plasma concentration before (pre) and after (post) *B. carinata* intervention with the preparation from raw *B. carinata* leaves (+ITC) as pg/mL −ITC: *n* = 18; +ITC: *n* = 20. Outlier removed. Minimum and maximum values, lower and upper quartiles, and median values are given. Asterisks indicate statistically significant differences (** *p* ≤ 0.01; Wilcoxon matched-pairs signed rank test).

Intervention with the preparation from cooked *B. carinata* leaves (−ITC) had no significant effect on the measured plasma PGE_2_ concentration (pre: 142 ± 46 pg/mL vs. post: 138 ± 40 pg/mL, Figure 5). Intervention with the preparation from raw *B. carinata* leaves (+ITC) significantly reduced the PGE_2_ concentration in the plasma of the subjects (pre: 162 ± 57 pg/mL vs. post: 135 ± 36 pg/mL).

## 4. Discussion

Previous in vitro research has already demonstrated that extracts from raw *B. carinata* leaves have anti-genotoxic potential against AFB1 [9]. However, in Ethiopia, where *B. carinata* is originally cultivated, as well as in other parts of Africa, it is mostly consumed as a cooked vegetable. In fact, typically, most of the *Brassicaceae* used as human foods are eaten following thermal processing (e.g., cooking, steaming, microwaving, frying, deep-frying). *Brassica* vegetables are well acknowledged for their health-promoting phytochemicals and it is widely assumed that the breakdown products of GLS, the ITC, are primarily responsible for the chemopreventive potential of the members of the *Brassicaceae* family [3]. As a consequence, extensive thermal processing that prevents formation of ITC via inactivation of the enzyme myrosinase [20] is considered to reduce health benefits of *Brassica* consumption. This hypothesis leads to the aim of the present intervention trial investigating whether the protective potential of *B. carinata* is also evident in vivo and whether this vegetable can be recommended being consumed raw or cooked for obtaining maximum health benefits.

The absence of AITC—the breakdown product of sinigrin and main ITC in *B. carinata*—in the preparation from cooked plant material indicated that the myrosinase enzyme was successfully inactivated by the 10 min cooking procedure. Accordingly, AITC metabolites were not detected in plasma and urine samples of the subjects that consumed the preparation from cooked *B. carinata* leaves except for low amounts of AITC-NAC detected in urine. This is most likely the result of the myrosinase-like activity of the gut microbiota, which has been well documented to convert sinigrin to AITC [29,30]. The high standard deviations of AITC metabolites observed in plasma and urine samples presumably reflect the inter-individual variation in biological response due to genetic and other factors that influence metabolism and ITC excretion as observed before [31,32].

Previous intervention and in vitro studies suggested that consumption of AITC-containing *Brassica* vegetables confer protection against carcinogenic action [33,34]. A recent in vitro study by our group on raw and processed *B. carinata* already indicated that anti-genotoxic and antioxidant activities are not limited to raw plant material [9]. Now, results of the present human intervention study rather show that consumption of *B. carinata* is able to strongly reduce the susceptibility of PBMC to DNA damage induced by AFB1 ex vivo, but this is evident only when the plant is cooked. This indicates that the anti-genotoxic effect of *B. carinata* is not primarily mediated by AITC.

Genotoxic effects of AFB1 in human cells result from transversion mutations in DNA via binding of metabolically activated AFB1 to guanine. Here, conversion into the active AFB1-8,9-epoxide by phase I enzymes of the liver xenobiotic metabolism (mainly CYP3A4) is highly relevant [35]. These epoxides create DNA adducts by binding to the guanine-N7 of dsDNA and thereby introduce a GC-TA transition [36]. Additionally, oxidative stress may be one of the underlying mechanisms for AFB1-induced cell injury and DNA damage. Activation of oxidative stress and pro-inflammatory pathways during AFB1-induced hepatocarcinogenesis has been shown [36]. Increased production of reactive oxygen species (ROS), surpassing the capacity of antioxidant mechanisms of defense, leaves cells vulnerable to oxidation processes of relevant cellular structures and paves the road to tumorigenesis [37]. Indeed, in an accompanying in vitro experiment to the present study, DNA damage induction by AFB1 could be blocked in PBMC by the addition of the antioxidant, NAC. This argues for a mechanism which is related to intracellular ROS. DNA damage induced by S9 activated AFB1 could be reduced, though not completely blocked, by NAC addition. Thus, the genotoxicity of activated AFB1 is most likely mediated by a combination of oxidative stress and other mechanisms.

Besides ITC, polyphenols have been reported to possess chemopreventive properties [38], and the anti-genotoxic/antioxidant activity of *Brassicaceae* has also been linked to polyphenols [39]. It has been shown before that the polyphenol reaction products formed during the cooking process could compensate the decay of the parent compounds´ antioxidant activity [40]. Bound phenolic compounds can be released from the plant cell walls during the heating processes, increasing the antioxidant capacity of the plant material. Especially, hydroxycinnamic acids, such as ferulic and caffeic acid derivatives, are frequently bound to cell walls, as they provide stability via crosslinking of cell wall polymers [41]. Deacylation and deglycosylation of *Brassica* polyphenolic compounds has been previously described in *B. carinata* [9] and *B.oleracea* [40] while caffeic acid derivatives are less responsive to cooking. Another important process, which happens during thermal treatment, is the break-down of larger compounds, such as flavonoids, to smaller phenolic acid derivatives, which have a higher antioxidant capacity [42]. Increased plasma TAC has been associated with a high consumption of fruits and vegetables, and seems to reflect the levels of individual antioxidants in plasma [43,44]. Here, the TAC in plasma of participants was not affected by the intervention with *B. carinata*, neither with raw nor cooked preparations. A possible explanation is that, although raw and cooked *B. carinata* leaves differ in their polyphenol composition, the cooking procedure did not significantly change the total polyphenol content. However, there are limits to the TAC concept that should be considered when interpreting ‘no effect’ reports [45]. We cannot exclude, for example, that the presence of other antioxidants, including vitamins, which might easily surpass the plasma concentration of polyphenols, play a role in masking potential effects of *B. carinata* polyphenols on TAC.

Just like oxidative stress, persistent inflammatory stimuli can contribute to the development and progression of chronic diseases, including cancer, and, in fact, are often closely linked. Reduction of pro-inflammatory mediators is thus an important part of chemoprevention. A decrease in inflammatory mediators has been described for ITC in general and AITC in particular before, primarily through inhibition of NFκB and blocking of the COX-2 signaling pathway [4,46]. PGE_2_, a major metabolite produced by COX-2, is one of the best studied eicosanoids that contributes to inflammation [47]. Under physiological conditions, PGE_2_ mediates versatile biological activities, such as the regulation of immune responses, blood pressure, or gastrointestinal integrity. During inflammation, it is involved in processes leading to the classic signs, such as redness or pain [48]. In the present trial, plasma levels of the inflammation marker PGE_2_ were significantly lower after intervention with the AITC-containing *B. carinata* preparation, but not after intervention with the cooked material. In this respect, these results meet the expectations suggesting that AITC is very likely responsible for the anti-inflammatory properties of *B. carinata*.

This is not the only study reporting thermal processing being not solely detrimental to the beneficial effects of vegetable consumption, but, on the contrary, increases expected health benefits. The extent of plant compound loss or retention during thermal processing, however, depends on the type of thermal treatment for food processing, e.g., stir-frying, boiling, microwaving, or deep-frying [19,49]. As mentioned earlier in the text, evidence suggests that short-term cooking of some vegetables increases the bioactivity of phenolic compounds, e.g., in terms of an increased antioxidant capacity [50,51]. Unrelated to the phytochemical composition of plant foods, the effects of thermal processing on the prevalence of bacterial or parasitic infection associated with consumption of raw vegetables should not be neglected [52,53].

One limitation of the study was that participants were blinded, but because the plant preparations were slightly different in their taste (pungent/bitter taste of AITC) and color it cannot be excluded that some participants were able to guess which trial arm was the active intervention. In general, true blinding is challenging to achieve in food interventions, and is only achievable with few foods. Another point is that about three times more women than men conducted the study. The study recruitment was open to both males and females and gender balance was not a requirement in our study design. The literature is not clear about potential gender effects on the basal DNA damage according to previous studies [54,55] as determined by the comet assay and did not play a relevant role for our research hypothesis. Also, with an average age of 22.7 years, the enrolled participants were of a quite young age, which is likely due to the way that we advertised the study. Garm et al. [55] could not find age effects on DNA damage, but on DNA repair. Thus, in our opinion, the data cannot be translated to the elderly population.

## 5. Conclusions

In the present human intervention trial, cooking was not shown to be a mitigating factor for the anti-genotoxic properties of the plant despite the absence of AITC. Quite the contrary was true. However, the presence of AITC seems to be relevant for anti-inflammatory capacity, which has been studied here as a secondary parameter. Consequently, to fully exploit the complexity of the health-promoting potential of Ethiopian kale—and possibly other *Brassica* species—a mix of both raw and cooked vegetables should be part of the diet. It needs to be emphasized here that the protective effects were observed with amounts reflecting a normal, realistic dietary intake. The results support previous in vitro data and thus sustain the rationale for promotion of African leafy vegetable cultivation in Eastern Africa as a measure to combat effects of unavoidable aflatoxin exposure. The observed effects should be further investigated in long-term applications, also with regard to food hygiene and further adverse and beneficial compounds.

## Figures and Tables

**Table 1:** AITC metabolites detected in plasma and urine of subjects (*n* = 22) 2 h post-intervention.

			Plasma	Urine
**Cooked (−ITC)**	AITC-	GSH	n.d.	n.d.
		CysGly	n.d.	n.d.
		Cys	n.d.	n.d.
		NAC	n.d.	9.36 ± 9.81
	Total		0	9.36 ± 9.81
**Raw (+ITC)**	AITC-	GSH	53.90 ± 10.17	n.d.
		CysGly	233.07 ± 167.55	n.d.
		Cys	92.71 ± 71.811	n.d.
		NAC	23.32 ± 10.21	38.07 ± 21.00
	Total		403.00 ± 238.59	38.07 ± 21.00

Values are given in nmol/L plasma and µmol/L urine (means ± SD). n.d. = not detected. GSH: glutathione; CysGly: cysteinylglycine; Cys: cysteine; NAC: N-acetylcysteine; ITC: isothiocyanates; AITC: allyl ITC.

## Supplementary Materials

The following are available online at http://www.mdpi.com/2072-6643/10/11/1622/s1, Table S1: Phenolic compounds in μg/g dry mass of raw and cooked *B. carinata* leaves, Table S2: Special GLS/ITC-free and polyphenol-reduced diet.
