# Supplementary material for: Are Raw Brassica Vegetables Healthier Than Cooked Ones? A Randomized, Controlled Crossover Intervention Trial on the Health-Promoting Potential of Ethiopian Kale
Source: Nutrients. 2018 Nov 2;10(11):1622. doi: 10.3390/nu10111622 (PMC6266411; doi:10.3390/nu10111622)
Supplement: Supplementary file 1 [file nutrients-10-01622-s001.pdf]

Table S1. Phenolic compounds in µg/g dry mass of raw and cooked *B. carinata* leaves. Values in bold indicate statistical significant difference ($p \leq 0.05$).

	raw	cooked
Caffeoylquinic acid (chlorgensaeure)	**47±2**	**38±2**
5-p-coumaroylquinic acid	90±40	43±5
Quercetin-3-O-sophoroside-7-O-D-glucoside	49±20	38±28
p-coumaroyl-glycoside	211±123	156±122
Kaempferol-3-O-sophoroside-7-O-D-glucoside	**123±117**	**473±131**
Kaempferol-3-O-sophoroside-7-O-diglucoside	27±25	66±9
Isorhamnetin-3-O-D-glucoside-7-O-D-glucoside	184±91	132±29
Kaempferol-3-O-hydroxyferuoyl-sophoroside-7-O-D-diglucoside	411±86	347±31
Kaempferol-3-O-hydroxyferuoyl-sophoroside-7-O-D-glucoside	756±157	639±57
Kaempferol-3-O-caffeoyl-sophoroside-7-O-D-glucoside	308±41	160±129
Isorhamnetin-3-O-hydroxyferuloyl-sophoroside-7-O-glucoside	124±46	219±173
Isorhamnetin-3-O-caffeoyl-sophoroside-7-O-diglucoside	129±82	119±24
Isorhamnetin-3-O-hydroxyferuloyl-sophoroside-7-O-diglucoside	191±26	235±139
Kaempferol-3-O-D-glucoside-7-O-D-glucoside	162±202	54±38
Kaempferol-3-O-sinapoyl-sophoroside-7-O-diglucoside	326±150	197±33
Kaempferol-3-O-feruloyl-sophoroside-7-O-diglucoside	**280±45**	**77±6**
Isorhamnetin-3-O-caffeoyl-sophoroside-7-O-D-glucoside	773±123	1058±210
Kaempferol-3-O-sinapoyl-sophoroside-7-O-D-glucoside	457±41	280±180
Kaempferol-3-O-feruloyl-sophoroside-7-O-D-glucoside	36±5	190±278
Kaempferol-3-O-coumaroyl-sophoroside-7-O-D-glucoside	52±4	166±157
Isorhamnetin-3-O-sinapoyl-sophoroside-7-O-glucoside	67±8	63±2
Isorhamnetin-3-O-feruloyl-sophoroside-7-O-glucoside	29±4	71±62
Isorhamnetin-3-O-coumaroyl-sophoroside-7-O-glucoside	55±9	66±4
Isorhamnetin-3-O-sophoroside	21±0	21±0

Kaempferol-3-O-hydroxyferuloyl-sophoroside	**25±8**	**9±4**
Isorhamnetin-3-O-sophoroside	21±0	21±0
Kaempferol-3-O-caffeoyl-sophoroside	4±0	4±0
Kaempferol-3-O-sophoroside7-sinapoyl-glucoside	84±88	77±19
Kaempferol-3-O-sinapoyl-sophoroside	4±0	4±0
Kaempferol-3-O-feruloyl-sophoroside	4±0	4±0
Quercetin-3-O-disinapoyl-triglucoside-7-O-D-glucoside	**221±19**	**172±8**
Kaempferol-3-O-disinapoyl-triglucoside-7-O-D-glucoside	**373±37**	**268±5**
Disinapoyl-gentiobioside	**77±3**	**46±0**
Sinapoyl-feruloyl-gentiobiose	**65±4**	**43±1**
Trisinapoyl-gentiobioside	41±10	34±3
Disinapoyl-feruloyl-gentiobiose	36±8	31±0

Table S2. Special GLS/ITC-free and polyphenol-reduced diet.

A: Vegetables and fruits to be avoided during wash out period

Vegetable or fruit	Scientific name
Leaf mustards	*Brassica carinata, B. juncea, B. perviridis, B. narinosa, B. nigra, B. campestris*
Cauliflower	*Brassica oleracea* var. *botrytis*
Broccoli	*Brassica oleracea* var. *italica*
Watercress	*Nasturtium officinale*
Chinese cabbage	*Brassica rapa* ssp. *pekinensis*
Garden cress	*Lepidium sativum*
Kale	*Brassica oleracea* var. *sabellica*
Herb turnip	*Brassica rapa* ssp. *rapa*
Caper	*Capparis spinosa*
Nasturtium	*Tropaeolum* L.
Kohlrabi	*Brassica oleracea* var. *gongylodes*
Turnip	*Brassica napus* ssp. *rapifera*
Cabbage	*Brassica oleracea* var. *capitata*
White turnip	*Brassica rapa* ssp. *rapa*
Horseradish	*Armoracia rusticana*
Mizuna	*Brassica rapa* var. *japonica*
Green Chinese cabbage (Pak Choi)	*Brassica rapa* ssp. *chinensis*
Papaya	*Carica papaya*
Radish	*Raphanus sativus* var. *sativus*
Rape (rape seed or oil or leaves)	*Brassica napus*
Bunch or Japanese radish	*Raphanus sativus* var. *niger*
Brussels sprouts	*Brassica oleracea* var. *gemmifera*
Red cabbage	*Brassica oleracea* var. *capitata* var. *rubra*
Turnip greens	*Brassica rapa* var. *rapa*
Rocket (broad, thick leaf)	*Diplotaxis muralis*
Rocket (arugula)	*Eruca sativa* (früher: *Rucola coltivata*)
Rocket (narrow-leaved, wild rocket)	*Diplotaxis tenuifolia*
Mustards	*Sinapis* L.
White cabbage	*Brassica oleracea* var. *capitata* var. *alba*
Savoy cabbage	*Brassica oleracea* var. *sabauda*

B: Permitted meals during the intervention period

Foods

White flour bread or buns
Pasta (durum wheat)
Rice, semolina, barley
Potatoes (peeled) and potato products
Meat and Fish
Eggs (maximum 2 per day)
Sausage (moderate)
Cheese (moderate), fresh cheese
Quark, yoghurt (natural)
Honey
Spices (moderate)
Cucumber (peeled)
Pretzel salt sticks
Rice waffles
Butter cookies

Drinks

Water
250ml coffee or tea per day (= approx. 2 cups or 1 mug; also decaffeinated coffee counts!)
Sodas (not caffeinated, without added vitamins)
Milk (moderate)
